# Coeliac disease: the showcase of interaction between genes, environment and immunology in chronic tissue inflammation

**DOI:** 10.1186/1479-5876-9-S2-I1

**Published:** 2011-11-23

**Authors:** Ludvig M Sollid

**Affiliations:** 1 Centre for Immune Regulation, Institute of Immunology, University of Oslo and Oslo University Hospital, Oslo, Norway

## 

Coeliac disease is a multifactorial disease controlled by multiple genes and environmental factors. Gluten proteins of grains are a key environmental factor as the disease goes in compete remission when gluten proteins are eliminated from the diet of the patients. The disease can be categorised as both a food hypersensitivity disorder as well as an autoimmune disease. Autoantibodies to the enzyme transglutaminase 2 (TG2) typical of coeliac disease have higher disease specificity and sensitivity than autoantibodies for any other disorder. Both the autoantibodies and the disease lesion of the small intestine, characterised by villous blunting, lamina propriaplasmacytosis and intraepithelial lymphocytosis, are reversibly dependent gastrointestinal exposure to cereal gluten proteins. There is strong association of coeliac disease with HLA-DQ2.5 and HLA-DQ8, and the presence of antibodies to TG2 is also completely dependent on carriage of DQ2.5 or DQ8. In addition to HLA genes, genome wide association studies have so far identified 39 non-loci implicated in the disease. Most of the coeliac disease susceptibility genes are related to the function of antigen presenting cells and T cells, and many risk genes are shared with organ specific autoimmune diseases.

Research in our laboratory has demonstrated that gluten reactive CD4 T cells can be found in the lamina propria of coeliac disease patients, but not healthy controls. These T cells recognise proline-rich gluten peptides that have become deamidated by TG2 in the context of DQ2.5 or DQ8 suggesting that preferential antigen presentation of as the reason for the HLA association. DQ2.5 and DQ8 is necessary but not sufficient for coeliac disease development, suggesting that activation of gluten reactive CD4 T cells is a key step in the pathogenesis. Many similar, yet distinct T-cell epitopes exist. The selection of gluten T-cell epitopes depends on gastrointestinal proteolysis, TG2 specificity and HLA peptide binding. Intraepithelial CD8 that bear NK cell receptors are involved in killing of stressed enterocytes. Although the mechanisms still have to be elucidated, this process seems to involve the gluten reactive CD4 T cells. A strong anti-gluten T cell response require high HLA binding stability of gluten peptides, and the disparate HLA association between two closely related HLA molecules, HLA-DQ2.5 and HLA-DQ2.2, seems to relate to this phenomenon. Finally, among the plasma cells of the lesion, many of them (10%) are specific for TG2. The TG2 specific plasma cells utilise a limited repertoire of variable genes with low degree of somatic mutation. Thus this antibody response, despite being part of a chronic condition, bears signs of a primary response. Together, these findings highlight that coeliac disease offers unique opportunities for dissection of T cell and B cell responses that lead to an autoimmune disease.

